# Protein succinylation associated with the progress of hepatocellular carcinoma

**DOI:** 10.1111/jcmm.17507

**Published:** 2022-10-29

**Authors:** Wenhui Bai, Li Cheng, Liangkun Xiong, Maoming Wang, Hao Liu, Kaihuan Yu, Weixing Wang

**Affiliations:** ^1^ Department of Hepatobiliary Surgery, Eastern Campus Renmin Hospital of Wuhan University Wuhan China; ^2^ Department of Intensive Care Unit, Eastern Campus Renmin Hospital of Wuhan University Wuhan China; ^3^ Department of Hepatobiliary Surgery Renmin Hospital of Wuhan University Wuhan China

**Keywords:** hepatocellular carcinoma, model, prognosis, succinylation

## Abstract

Although post‐translational modification is critical to tumorigenesis, how succinylation modification of lysine sites influences hepatocellular carcinoma (HCC) remains obscure. 90 tumours and paired adjacent normal tissue of liver cancer were enrolled for succinylation staining. 423 HCC samples with 20 genes related to succinylation modification from TCGA were downloaded for model construction. Statistical methods were employed to analyse the data, including the Non‐Negative Matrix Factorization (NMF) algorithm, t‐Distributed Stochastic Neighbour Embedding (t‐SNE) algorithm, and Cox regression analysis. The staining pan‐succinyllysine antibody staining indicated that tumour tissues had a higher succinyllysine level than adjacent tissues (*p* < 0.001), which could be associated with a worse prognosis (*p* = 0.02). The survival was associated with pathological stage, tumour recurrence status and succinyllysine intensity in the univariate or multivariable cox survival analysis model. The risk model from 20 succinyllysine‐related genes had the best prognosis prediction. The high expression of succinylation modification in HCC contributed to the worse patient survival prognosis. Model construction of 20 genes related to succinylation modification (MEAF6, OXCT1, SIRT2, CREBBP, KAT5, SIRT4, SIRT6, SIRT7, CPT1A, GLYATL1, SDHA, SDHB, SDHC, SDHD, SIRT1, SIRT3, SIRT5, SUCLA2, SUCLG1 and SUCLG2) could be reliable in predicting prognosis in HCC.

## 1 INTRODUCTION

The liver cancer screening of high‐risk populations is helpful for diagnosis, prognosis and early treatment, which is important to improve the longevity of patients.^1^ In China, high‐risk factors of liver cancer mainly include hepatitis B virus (HBV) infection, hepatitis C virus (HCV) infection, excessive drinking, non‐alcoholic steatohepatitis, long‐term consumption of contaminated foods with aflatoxin, liver cirrhosis caused by other reasons, and the person with a family history of liver cancer, especially men over 40 years of age.^2^ At present, serum AFP is a commonly used and significant indicator for the diagnosis of liver cancer. Due to the lack of prognostic markers for hepatocellular carcinoma (HCC), we need to investigate other biomarkers further, either alone or in combination with other methods, to assess HCC survival and tumour regression.

Hepatic glycogen is crucial to regulating blood glucose concentration to maintain its stability.^3^ When labor, hunger, fever, and blood sugar are consumed in large quantities, liver cells can decompose liver glycogen into glucose to enter the blood circulation. Therefore, under normal circumstances, the synthesis and decomposition of liver glycogen often maintain a dynamic balance.^4^ Anaerobic metabolism accounts for only 2% of the total energy in a healthy body at rest, but 50% of their energy from anaerobic glycolysis is provided for malignant tumour cells, and oxygen can no longer inhibit glycolysis. With an in‐depth investigation of various metabolic pathways in living organisms, the amount and activity of enzymes involved in catalysing various reactions in the metabolic pathway can not only determine the size but also change the direction of the metabolic flux.^5^ Succinate dehydrogenase (SDH) is the only enzyme in the tricarboxylic acid cycle (TCA) incorporated into the inner mitochondrial membrane.^5^ In addition to catalysing the third redox reaction in the TCA cycle, SDH enables flavin adenine dinucleotide (FADH2) produced by dehydrogenation of succinate to be transferred to the iron–sulfur center of the enzyme before oxidation and release of energy in the respiratory chain.^5^ Research has proved that the specific activity of SDH in liver cancer cells is 42% lower than that of normal hepatocytes. In addition, the significant reduction in SDH activity inevitably affects the TCA cycle, leading to the accumulation of succinate.^6^

Succinate is known as a key substrate for succinylation modifications. As an effective protein modification method commonly adopted in all kinds of prokaryotes and eukaryotes, succinylation plays an essential role in various life activities.^7^ So far, limited studies have demonstrated the association between succinylation modification of lysine sites and the development of HCC. According to previous studies, the succinylation of lysine residues in proteins participates in the multi‐level biological activities of the cell.^7^, ^8^ Lu found that Glutaminase (GLS) was highly expressed in pancreatic ductal adenocarcinoma. Compared with normal cells, the growth and survival of pancreatic ductal adenocarcinoma were more dependent on glutamine metabolism. Besides, a succinylation modification was also found on the glutaminase (GLS) protein, which occurred at the K311 position and could be mediated by succinyl‐CoA.^8^ The succinylation promoted the conversion of GLS from monomer to active tetramer, thereby improving its catalytic activity and enhancing the catabolism of glutamine. The research emphasized new mechanisms of small molecular metabolites that could regulate mitochondrial metabolic activity and cellular oxidative stress, thus providing insights into new strategies for tumour treatment.

We have reported the correlation between succinylation and HCC progress in current studies. In addition, via public data from TCGA, risk model construction with succinylation‐related genes validated the primary role of succinylation in predicting the development of HCC.

## 2 MATERIALS AND METHODS

### 2.1 Samplecollection

Ninety patients with hepatocellular carcinoma were recruited to the Renmin Hospital of Wuhan University from 2019 to 2021. 90 tumour and paired adjacent tissues from surgical hepatocellular carcinoma specimens were extracted for the following immunohistochemistry (IHC) staining. The hepatocellular carcinoma diagnosis followed standards for diagnosing and treating primary liver cancer (2021 edition).^9^ This study was conducted with the approval of the ethics committee of the Renmin Hospital of Wuhan University and written informed consent from each participant. All research on humans (individuals, samples or data) was performed under the principles stated in the Declaration of Helsinki.

Clinical data were grouped as follows: according to age, one group ≤65 years old and the other >65 years old; according to gender, male group and female group; from tumour size, one ≤5 cm and the other>5 cm; based on pathology grade, grade I, grade II and grade III; from T stage, T1, T2 and T3 groups; under AJCC staging, TNM1, TNM2, TNM3; from recurrence situation, recurrence and non‐recurrence group; according to liver cirrhosis status, cirrhosis and non‐cirrhosis; as per Hepatitis B virus infection status, positive and negative group.

### 2.2 Immunohistochemistry (IHC) staining and results interpretation

Anti‐Succinyllysine Rabbit pAb (PTM‐401) was purchased from PTM Bio, and the steps of immunohistochemistry staining shortly were as follows. After dewaxing and hydration, it was washed twice with PBS for 5 min each time. Fresh 3% H_2_O_2_ with distilled water or PBS was prepared and sealed at room temperature for 5–10 min before being washed with distilled water 3 times. Antigen retrieval was then conducted, and PBS was used for washing for 5 min. Normal goat serum blocking solution dropwise was added and kept for 20 min at room temperature. Afterwards, excess liquid was shaken off before adding primary antibody dropwise. It was then kept at room temperature for 1 h or 4°C overnight or 37°C for 1 h (4°C overnight and then 37°C for 45 min) before being washed three times with PBS for 2 min each time. Biotinylated secondary antibody dropwise was added, which would be preserved at 20–37°C for 20 min. Then, after adding reagent SABC dropwise, the liquid was kept at 20–37°C for 20 min. Next, it was washed 4 times with PBS for 5 min each time. After Haematoxylin counterstaining for 2 min, hydrochloric acid and alcohol differentiation was achieved. In the end, results were observed with microscopy. In IHC staining, negative and positive control was set. The negative control used a primary antibody diluent instead of the primary antibody, and the positive one adopted a PD‐L1 antibody (Rabbit mAb #13684, CST). The other experimental conditions were consistent and performed simultaneously (Figure S1). The standardization scheme of original experimental data was interpreted. Staining intensity scores were 0 points (negative), 0.5 points (<1), 1 point (1+), 1.5 points (<2), and 2 points (2+). Scores for staining positive rate were 0 points (negative), 1 point (1%–25%), 2 points (26%–50%), 3 points (515%–75%), 4 points (76%–100%). The total score was the product of the “staining intensity score” and the “staining positive rate score”. Regarding survival analysis grouping, the total score <2 corresponds to the low expression group, and the total score ≥2 refers to the high expression group.

### 2.3 Public data download and analysis

The Liver Hepatocellular Carcinoma (LIHC) data were downloaded from the public database of the Cancer Genome Atlas Program (TCGA, https://www.cancer.gov/tcga). 423 samples were enrolled, which included 373 tumour tissues and 50 adjacent tumour tissues, and RNA‐seq of 20 succinylation‐related genes were picked for further clustering. 10 enzymes related to succinylation modification (SIRT1, SIRT2, SIRT3, SIRT4, SIRT5, SIRT6, SIRT7, CPT1A, KAT5 and GLYATL1) and 10 succinate metabolizing enzymes (SDHA, SDHB, SDHC, SDHD, SUCLA2, SUCLG1, SUCLG2, CREBBP, OXCT1 and MEAF6) were selected for predicting succinylation level. Data preprocessing principle required as follows: (1) TCGA RNA‐seq data with fragments per kilobase million (FPKM) standardize was used for further analysis; (2) 20 succinylation‐related genes were filtered for clustering in all tumour samples except adjacent tumour tissues; (3) Samples without clinical information were excluded. The analysis pipeline included (1) comparison of the RNA‐seq level between LIHC cohorts (373 tumour tissues vs. 50 adjacent tumour tissues); (2) comparison of the RNA‐seq level between LIHC paired samples (50 pairs of tumour and adjacent tumour tissues); (3) NMF and t‐SNE clustering analysis; (4) comparison of clinical characteristics among different clusters; and (5) Cox step regression analysis and risk score calculation.

### 2.4 Statistical analysis

R software (https://www.r‐project.org/) was employed for major statistical analysis. One‐way anova with Kruskai‐Wallis statistics test was performed using GraphPad Prism version 6.04 for Windows, GraphPad Software, La Jolla, California, USA, www.graphpad.com. Univariable and multivariable cox regression was conducted via the R platform with the help of R packages (‘survival’, ‘survminer’ and ‘survivalROC’). R software (https://www.r‐project.org/) was used for most statistical analyses. R package ‘glmnet’ was employed for Lasso regression analysis, package ‘rms’ was used for drawing nomogram, and the aggregate function in the ‘VIM package was adopted to judge the missing data. Survival analysis was done by using the package ‘survival’. Non‐Negative Matrix Factorization (NMF), a state‐of‐the‐art feature extraction algorithm, is useful when many attributes are ambiguous or have weak predictability.^10^ By combining attributes, NMF can produce meaningful patterns, topics, or themes. Each feature created by NMF is a linear combination of original attributes with a set of coefficients, which help measure the weight of each attribute. Data classifying was done by using the t‐Distributed Stochastic Neighbour Embedding (t‐SNE) algorithm.^11^

## 3 RESULTS

### 3.1 IHC staining analysis for liver cancer patients

**FIGURE 1:**
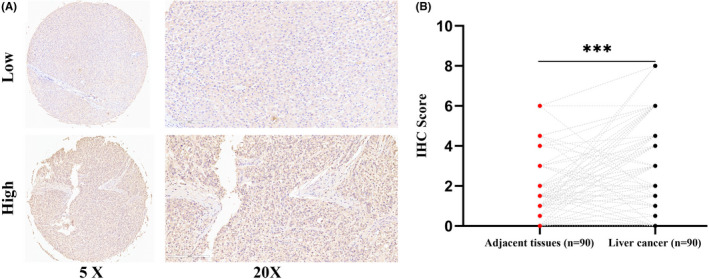
Immunohistochemistry staining of succinyllysine antibody in liver cancer tissues. (A) Comparison of succinyllysine scope between high and low staining scores. (B) Comparison of succinyllysine staining scores between Liver cancer and adjacent tissues

**FIGURE 2:**
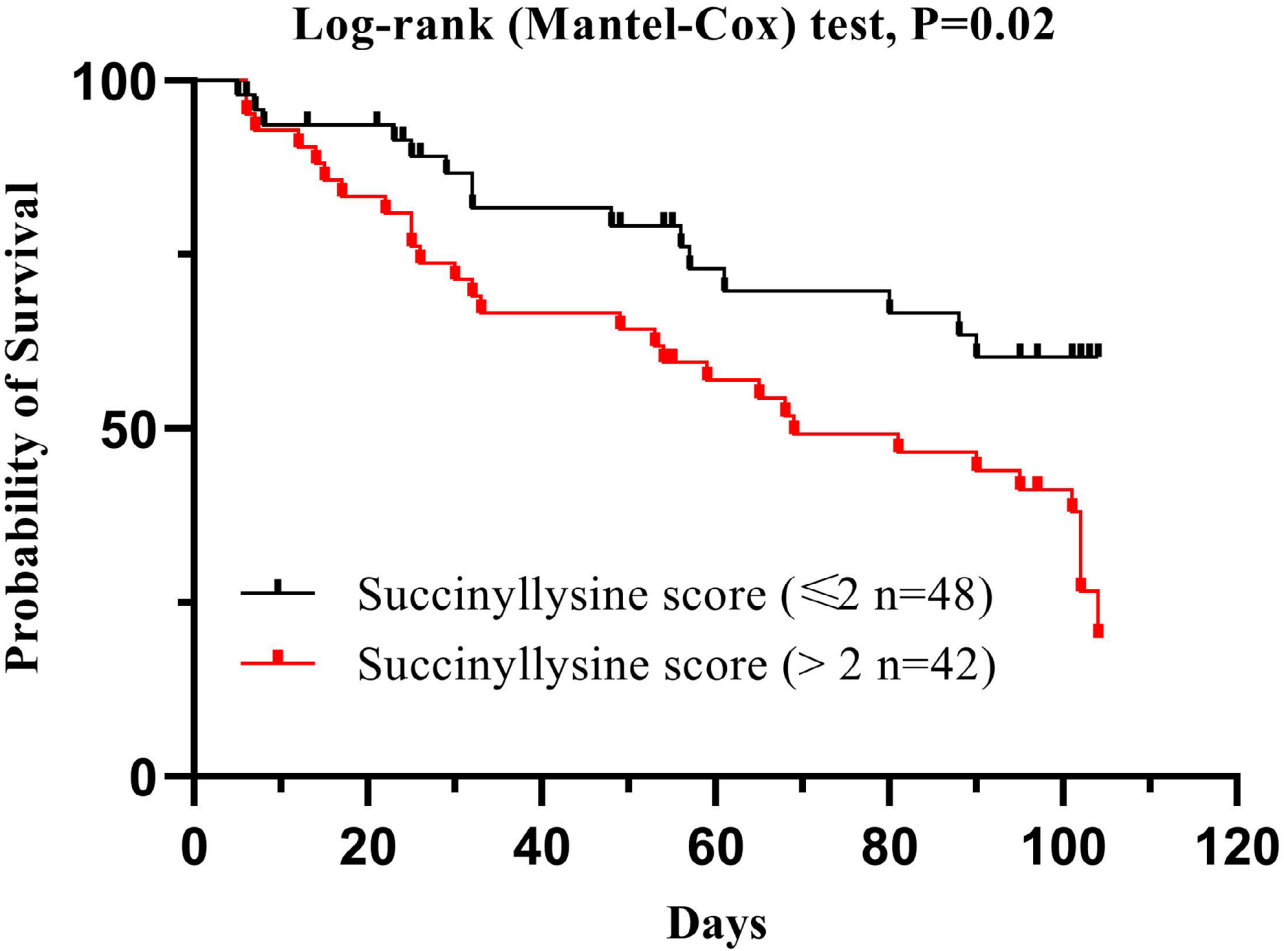
Survival analysis of succinyllysine antibody expression in cancer tissues

Ninety tumour and paired adjacent tissues received pan‐succinyllysine antibody IHC staining. 62 of 90 (68.89%) tumour tissues showed higher succinylation levels compared with adjacent tissues (25 of 90, 27.78%), and the difference was statistically significant (Table 1, *χ* ^2^ = 28.83, *p* < 0.001). IHC intensity score was calculated according to the instruction. Pair matched comparison demonstrated higher IHC scores in tumour tissues than in adjacent ones (Figure 1, *p* < 0.001). More importantly, according to survival analysis of succinylation antibody expression, a higher IHC score was associated with a worse prognosis (Figure 2, log‐rank test, *p* = 0.02).

**TABLE 1:** Differential expression of succinyllysine in liver cancer and adjacent tissues

	*n*	Succinyllysine expression		Chi‐square Value	*p* Value
		High (%)	Low (%)		
Liver cancer	90	62 (68.89%)	28 (31.11%)	28.83	0.000
Adjacent tissues	90	25 (27.78%)	65 (72.22%)		

Statistically significant (*p* < 0.05).

**FIGURE 3:**
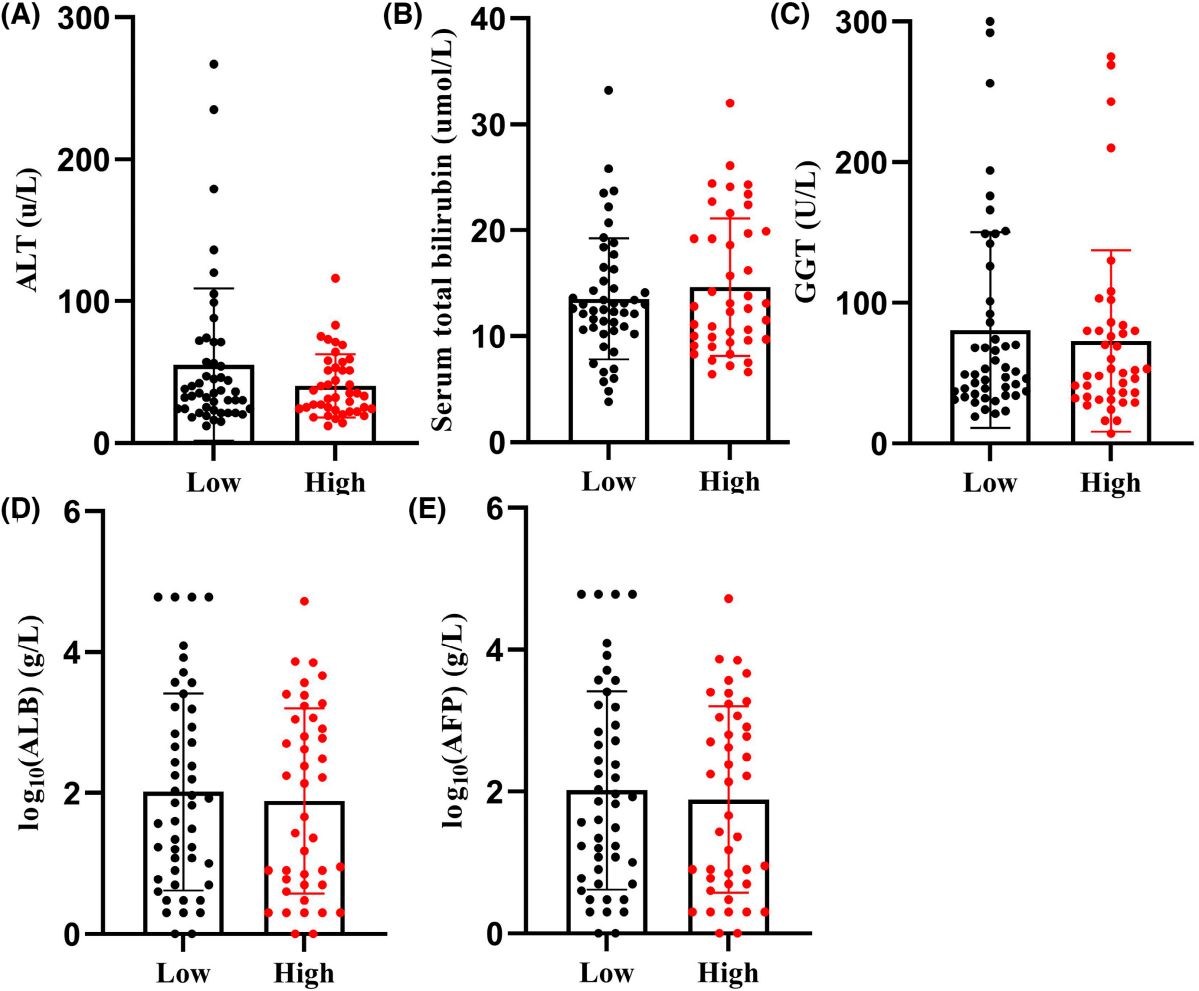
Comparison of clinical characterize of ALT (A), TBI (B), GGT (C), ALB (D) and AFP (E) between high and low succinyllysine antibody expression in cancer tissues

In addition, clinical characteristics, including age, sex, histological grades, T stage, AJCC stage, tumour recurrence, sclerosis, HBsAg level, and expression of PDL1 and CTLA4, were also analysed between patients with high and low IHC scores. No significant difference was found between them (Table 2). Clinical experiment test of liver function compared alanine aminotransferase (ALT), aspartate aminotransferase (AST), Gamma‐glutamyl transferase (GGT), Alpha Feta Globulin Protein (AFP), and Albumin (ALB) and Serum total bilirubin, which indicated no significant difference (Figure 3). Univariate and multivariable Cox survival analysis was done among clinical characteristics and pan‐succinylation IHC staining scores. Overall survival is associated with pathological stage, tumour recurrence status and succinylation intensity in the univariate and multivariable cox survival analysis model (Table 3). Age, sex, histological grade and tumour size did not influence the overall survival of liver cancer patients.

**TABLE 2:** Correlation between succinyllysine expression and clinicopathological characteristics

	Variables	Succinyllysine expression		Total	*χ* ^2^	*p* Value
		Low	High			
Age (year)	≤60	39	32	71	0.108	0.743
	>60	9	10	19		
Sex	Female	4	6	10	OR = 1.82	0.505^a^
	Male	44	36	80		
Grade	I/II	32	34	66	1.66	0.197
	III	16	8	24		
T stage	T1	34	29	63	0.1	0.998
	T2/T3	14	13	27		
AJCC stage	Ι	34	29	63	0.1	0.998
	II /III	14	13	27		
Recurrence	Yes	28	21	49	0.336	0.562
	No	20	21	41		
Sclerosis	No	4	5	25	OR = 1.48	0.729^a^
	Yes	44	37	56		
HBsAg	Positive	38	33	71	0.1	0.998
	Negative	10	9	19		
PDL1	Score ≥2	10	7	17	0.055	0.815
	Score <2	38	35	73		
CTLA4	Score ≥4	35	32	67	0.013	0.91
	Score <4	13	10	23		

Abbreviation: AJCC, American Joint Commission on Cancer.

^a^ Fisher exact test.

**TABLE 3:** Univariate and multivariate analyses of the factors correlated with overall survival of liver cancer patients

Variables	Univariate analysis			Multivariate analysis		
	HR	95% CI	*p* Value	HR	95% CI	*p* Value
Age	1	[0.99–1]	0.21			
Sex	0.56	[0.17–1.8]	0.34			
Grade	0.98	[0.54–1.8]	0.95			
Stage	2.3	[1.3–4]	0.0023	1.82	[1.0–3.3]	0.049
Size	1.1	[0.98–1.2]	0.16			
Recurrence	4.2	[2.2–8.2]	1.80E‐05	5.84	[2.74–12.47]	4.91E‐06
Succinyllysine	1.2	[1.1–1.4]	0.0073	1.29	[1.13–1.48]	0.00023

Statistically significant(*p* < 0.05).

### 3.2 Identifying liver cancer subtype based on the expression of succinylation‐related genes

**FIGURE 4:**
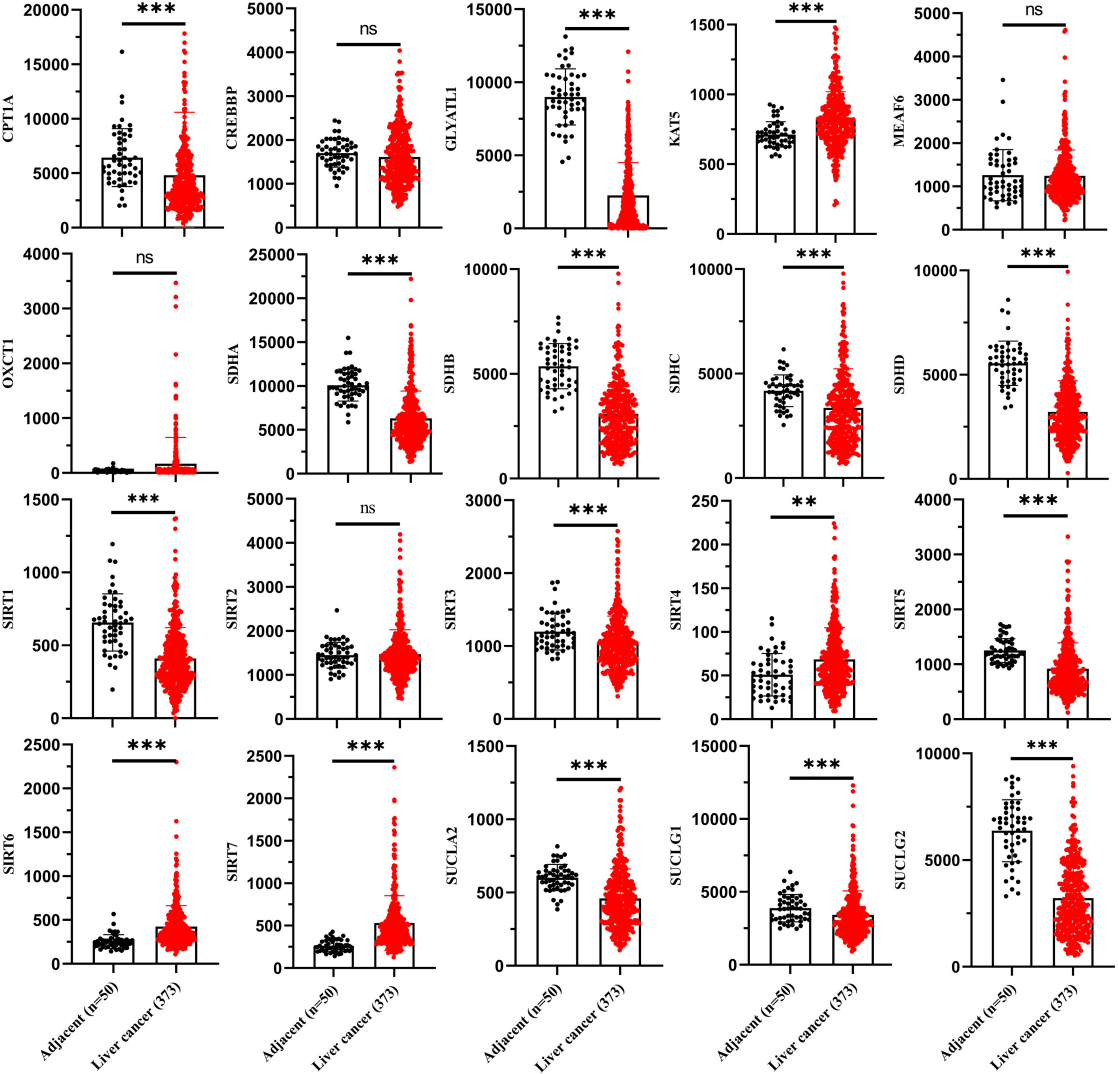
Comparison expression level of 20 succinylation modification related genes between liver cancer (*n* = 373) and adjacent tissues (*n* = 50) from TCGA database

**FIGURE 5:**
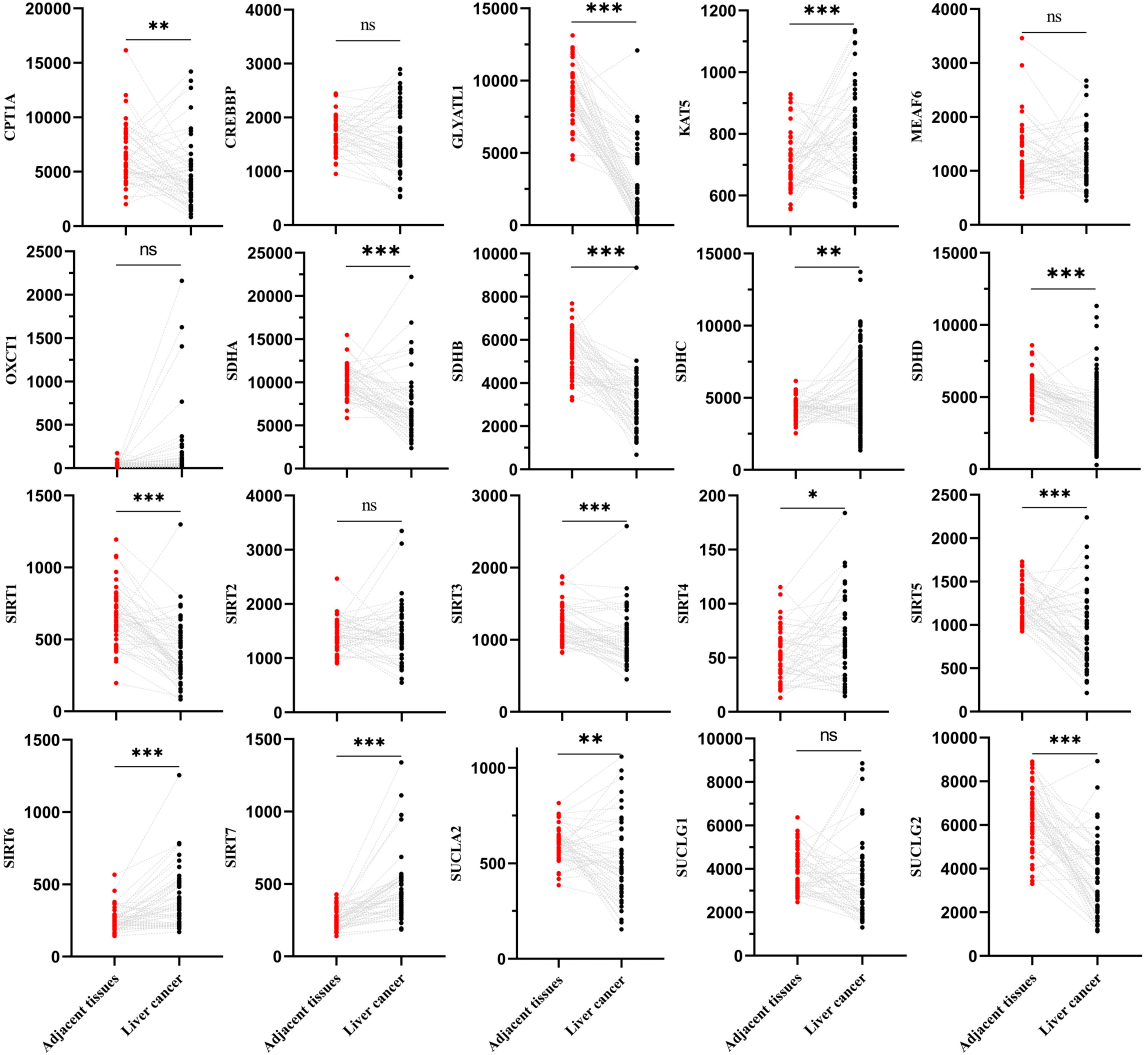
Comparison expression level of 20 succinylation modification related genes between 50 pairs of liver cancer and adjacent tissues from TCGA database

**FIGURE 6:**
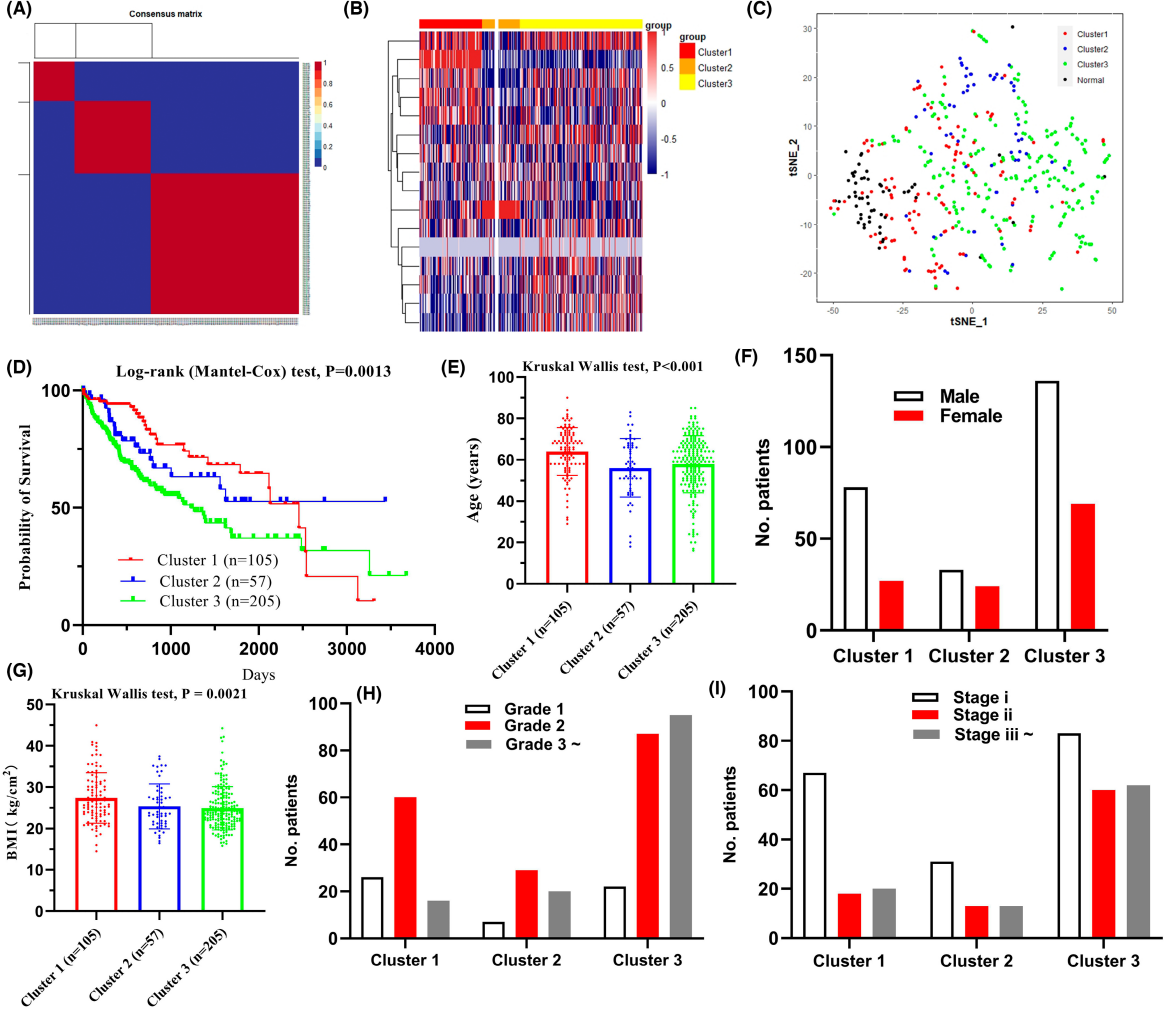
Subtype of liver cancer identification. (A) 373 Liver cancer samples were clustered into three types according to the consensus clustering matrix (k = 3). (B) Heatmap plot show 20 succinylation modification related genes were distributed in three clusters. (C) t‐SNE analysis of 373 Liver cancer and 50 adjacent samples based on expression levels of 20 succinylation modification related genes. (D) Survival analysis of three different clusters. Comparison clinical characteristic of age (E), sex (F), BMI (G), histological grade (H) and pathology stages (I) among three different clusters

Four hundred twenty‐three LIHC patients from the TCGA database and RNA‐seq expression levels from 20 succinylation‐related genes were downloaded for further clustering (Table S1). We compared the expression level of 20 succinylation‐related genes of tumours and adjacent tissues (Figure 4 and Figure 5). No significant difference between tumours and adjacent tissues was observed in MEAF6, OXCT1, SIRT2 and CREBBP. In terms of KAT5, SIRT4, SIRT6 and SIRT7, these four genes demonstrated higher expression in tumours than in adjacent tissues (Figure 4 and Figure 5). Twelve genes, CPT1A, GLYATL1, SDHA, SDHB, SDHC, SDHD, SIRT1, SIRT3, SIRT5, SUCLA2, SUCLG1 and SUCLG2, demonstrated lower expression in tumour than adjacent tissues (Figure 4 and Figure 5).Three clusters were identified using the NMF algorithm (Figure 6A), and parameter K = 3 showed the optimal number. The association matrix between gene expression and type of cluster was demonstrated in Figure 6B. T‐Distributed stochastic neighbour embedding (t‐SNE) analysis of all patients showed that patients in cluster 1 were next to normal tissues, but cluster 3 was apart from normal patients (Figure 6C).

Further prognostic survival analysis indicated patients in cluster 3 had the worst prognosis (Figure 6D, log‐rank test, *p* = 0.0013). In addition, age distribution led to differences among three clusters, and patients in cluster 3 were older than the others (Figure 6E, Kruskai‐Wallis statistics, *p* < 0.001). Sex and BMI distribution presented no difference among the three clusters (Figure 6F,G). High histological grades and pathology stages were noticed in cluster 3 patients (Figure 6H,I).

### 3.3 Risk model construction for twenty succinylation‐related genes

**FIGURE 7:**
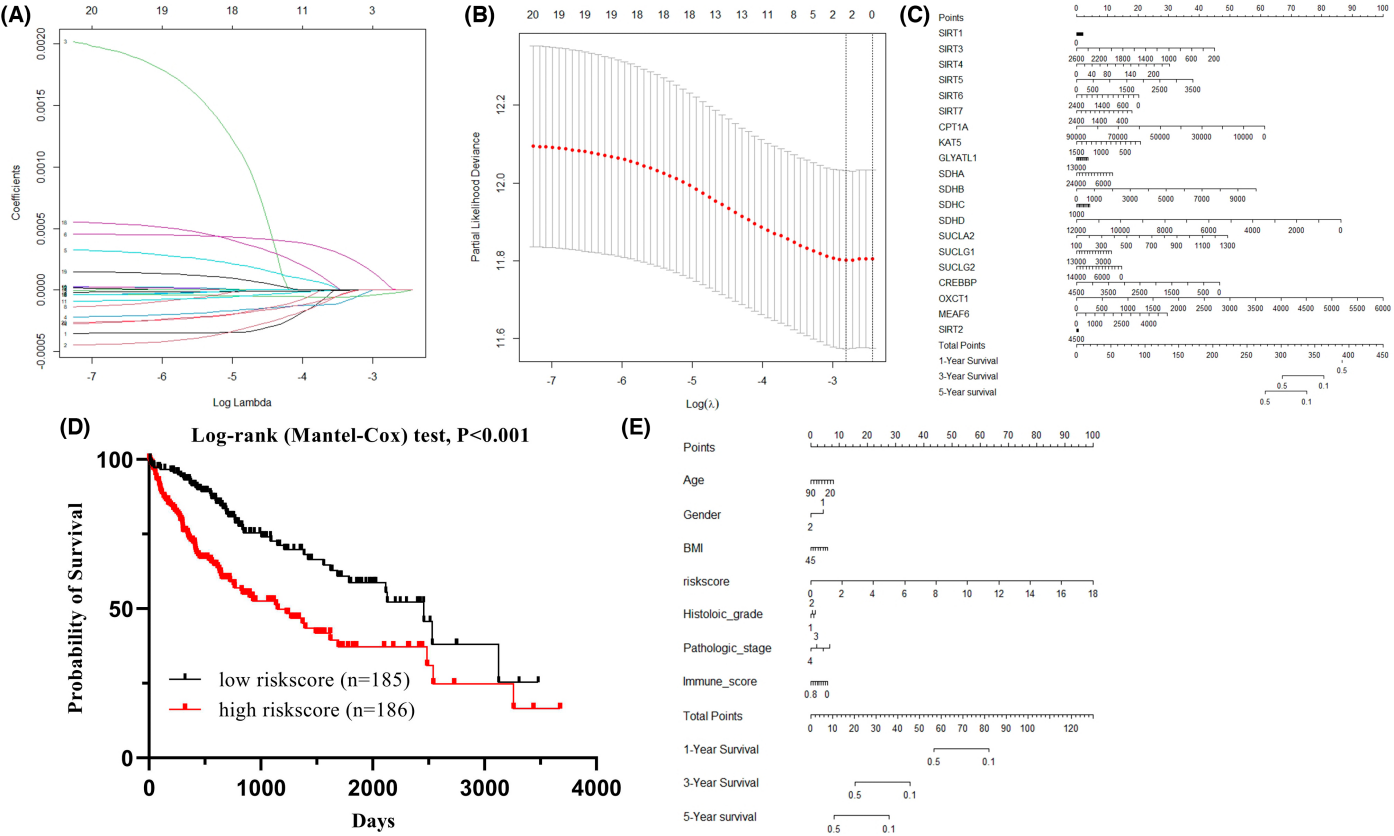
Lasso Cox regression model for signature gene analysing. (A) Trajectory trace of each independent variable. (B) The 95% confidence interval at different level of lambda. (C) Nomogram plot of 20 selected genes for predicting survival in liver cancer. (D) Survival analysis of risk scores in 373 cancer tissues. (E) Nomogram of clinical characterization for predicting survival in liver cancer

Risk model analysis was made using 20 succinylation‐related genes, and Lasso‐Cox regression analysis was constructed to filter significant signature genes. The optimal lambda was chosen based on independent variables, and eventually, λ = 0.064 was selected for calculation (Figure 7A). In addition, the 95% CI (confidence interval) at a different lambda level is shown in Figure 7B. The risk scores were extracted for further analysis, and the nomogram plot demonstrated a predicted prognosis in 1, 3 and 5 years (Figure 7C). In total, the risk score of each sample from expression data of 20 succinylation‐related genes was the best markers for predicting prognosis. More apparently, patients with a higher risk score got a worse prognosis (Figure 7D, log‐rank test, *p* < 0.001, Figure 7E and Table 4). Therefore, the risk model from succinylation‐related genes showed the best prognosis predicting effects.

**TABLE 4:** Univariate and multivariate analyses of the clinical characterize correlated with Overall survival of liver cancer patients in TCGA database

Variables	Univariate analysis			Multivariate analysis		
	HR	95% CI	*p* Value	HR	95% CI	*p* Value
Age	1	[1]	0.089			
Gender	1.2	[0.84–1.7]	0.31			
BMI	0.98	[0.94–1]	0.2			
Risk score	2.2	[1.6–2.9]	1.1E‐07	1.93908	[1.42–2.65]	0.0000322
Histological grade	1.1	[0.87–1.4]	0.44			
Pathological stage	1.7	[1.4–2.1]	1.20E‐07	1.55749	[1.27–1.91]	2.43E‐05
Immune score	0.65	[0.032–13]	0.78			

Statistically significant (*p* < 0.05).

## 4 DISCUSSION

In the current investigation, high levels of succinylation modification were often found to occur in patients suffering from liver cancer and were associated with prognosis. We provided evidence that genes related to succinylation modification contributed to the disease prognosis and could explain the heterogeneity of pathology in HCC patients. Moreover, this study warrants exploring strategies to increase succinylation and disease progression. However, the mechanism behind high levels of succinylation modification and HCC progress remains to be studied further.

The active center of an enzyme protein is necessary for performing its normal catalytic function.^12^ By comparing with the annotations of the database UniProt, Park et al. found that among the detected protein succinylation sites, 16 succinylation sites appeared in the cofactor binding region or enzyme catalytic region and that 74 succinylation sites existed around the active site of the enzyme,^13^ suggesting that the succinylation of protein lysine residues was involved in the enzyme's catalytic function. As mentioned above, SIRT5 could inhibit the enzyme activity of the pyruvate dehydrogenase complex and the complex enzyme body II (SDH) in the respiratory chain by reducing the succinylation of lysine.^14^ In addition, it was indicated that lysine succinylation affected the activity of these two enzymes. Disturbances in the pyruvate dehydrogenase complex activity were associated with human type II diabetes and other diseases. The activity‐related succinylation modification may provide new research directions on drug targeting.^14^ Isocitrate dehydrogenase can catalyse the formation of α‐ketoglutarate from isocitrate, and mutations in its succinylation sites K199 and K242 will reduce its activity.^15^, ^16^, ^17^ Two of the five catalytic sites of this enzyme have been identified as succinylation modification sites. There is a certain connection between succinylation and changes in catalytic products. 293 T cells expressed normal superoxide dismutase, and the active oxygen components in the cells were reduced by 19%.^18^ If the K123 site was desuccinylated, the active oxygen component in the cell would decrease by 43%. In other words, succinylation inhibited the activity of the SOD enzyme and affected its function.^18^ On the contrary, its ability to remove cellular reactive oxygen species would increase. The succinylation of methylglutaryl‐CoA synthase 2 modified its substrate binding region, causing its enzymatic activity to be negatively regulated and eventually hindering the production of ketone bodies.^19^ Pyruvate kinase PKM2 exerts the rate‐limiting enzyme function of glycolysis, and its succinylation of K498 affects the level of ROS in tumour cells.^17^, ^20^ Studies have shown that a variety of post‐translational modifications regulate the activity and function of PKM2.^17^, ^20^ Succinylation of K311 in 7 succinylation sites has been detected to promote the formation of PKM2 dimers and inhibit its tetramers, which can increase the protein kinase activity that phosphorylates the T11 of histone H3 and inhibit its catalytic activity using phosphoenolpyruvate as a substrate.^20^, ^21^ PKM2 can work with H1F1α to regulate white blood cells in macrophages activated by lipopolysaccharide.^18^ Its succinylation promotes the production of IL‐1β and other pro‐inflammatory cytokines, thus increasing inflammatory bowel disease susceptibility.^18^ In short, succinylation regulates various metabolic pathways by reversibly modifying various enzymes, which dynamically changes the enzyme activities, thus regulating different metabolisms according to the body's needs.^20^, ^21^, ^22^ Related discovery of succinylation in tumour cells or macrophages also fully demonstrates that this post‐translational modification method is inevitable with various diseases in the normal function of the body and immune system.^20^, ^23^

According to the enrichment analysis of lysine succinylated proteins detected in various organisms based on GO and KEGG databases, the modified proteins in mouse cells are mostly concentrated in keto acid metabolism, redox reaction, coenzyme metabolism and translation process.^24^ In general, 37 out of 51 proteins in the degradation pathway of valine, leucine and isoleucine were lysine succinylated, and 80% and 60% of the proteins involved in the tricarboxylic acid cycle and fatty acid metabolism, respectively. Most of the 261 lysine succinylated proteins identified in the germinated rice germ got involved in stress response, translation and sugar metabolism.^25^, ^26^, ^27^ We further enriched and analysed most proteins involved in ribosomes, tricarboxylic acid cycle, fatty acid metabolism, glycolysis, gluconeogenesis, pyruvate metabolism, oxidative phosphorylation, glyoxylic acid and dicarboxylic acid metabolism.^7^, ^25^, ^26^, ^27^ Among 204 lysine succinylated proteins of bacillus subtilis, 17% were involved in the translation process, 14% in sugar metabolism, and 11% in amino acid metabolism.^7^, ^25^, ^26^, ^27^ 462 lysine succinylations in the protein interaction network made by cytoscape were involved in the special protein secretion system, toxicity, adaptability and other functions of the bacteria.^7^, ^25^, ^26^, ^27^ In almost every metabolic process, certain proteins undergo lysine succinylation, indicating that lysine succinylation plays an important role in cells.^7^, ^25^, ^26^, ^27^

We also found that the disease modifier pathological succinylation was independent of changes in mtDNA copy number and levels of TCA cycle metabolites.^28^, ^29^ This finding suggests a possible multi‐target approach of addressing HCC pathology and activating SIRT5 activity, for example, through membrane‐permeable succinate to bypass the failure of producing succinate in the TCA cycle. Beyond the relevance for HCC, this study proved that the non‐physiological increase in reactive carbon species contributed to cellular dysfunction and disease through post‐translational modification. Tong found a succinylation modification on the glutaminase (GLS) protein occurred at the K311 position and was mediated by succinyl‐CoA, which could promote the conversion of GLS from monomer to active tetramer, thereby improving its catalytic activity and enhancing the catabolism of glutamine in pancreatic ductal adenocarcinoma.^8^ Chen revealed a novel role of SIRT5 in inhibiting peroxisome‐induced oxidative stress, in liver protection, and in suppressing HCC development.^30^

In conclusion, a high expression of succinylation modification level in HCC would result in a worse patient survival prognosis. Model construction of 20 succinylation modification‐related genes could be reliable in predicting prognosis in HCC (MEAF6, OXCT1, SIRT2, CREBBP, KAT5, SIRT4, SIRT6, SIRT7, CPT1A, GLYATL1, SDHA, SDHB, SDHC, SDHD, SIRT1, SIRT3, SIRT5, SUCLA2, SUCLG1 and SUCLG2).

## AUTHOR CONTRIBUTIONS

**Wenhui Bai:** Conceptualization (equal); data curation (equal); funding acquisition (equal); software (equal); supervision (equal); visualization (equal). **Li Cheng:** Conceptualization (equal); data curation (equal); funding acquisition (equal); investigation (equal); methodology (equal); supervision (equal); validation (equal); writing – review and editing (equal). **Liangkun Xiong:** Conceptualization (equal); data curation (equal); funding acquisition (equal); investigation (equal); project administration (equal); supervision (equal); validation (equal); writing – original draft (equal); writing – review and editing (equal). **Maoming Wang:** Conceptualization (equal); data curation (equal); investigation (equal); project administration (equal); validation (equal); writing – original draft (equal); writing – review and editing (equal). **Hao Liu:** Conceptualization (equal); data curation (equal); formal analysis (equal); investigation (equal); methodology (equal); supervision (equal); validation (equal); writing – original draft (equal); writing – review and editing (equal). **Kaihuan Yu:** Conceptualization (equal); data curation (equal); funding acquisition (equal); investigation (equal); software (equal); validation (equal); visualization (equal); writing – original draft (equal); writing – review and editing (equal).

## CONFLICT OF INTEREST

The authors declared that they have no competing interests.

## Supporting information


Figure S1

Table S1

## Data Availability

The data that support the finding of this article are available from the corresponding author upon reasonable request.
