# Supplementary figures and images for: Protein succinylation associated with the progress of hepatocellular carcinoma
Source: J Cell Mol Med. 2022 Oct 29;26(22):5702–12. doi: 10.1111/jcmm.17507 (PMC9667522; doi:10.1111/jcmm.17507)

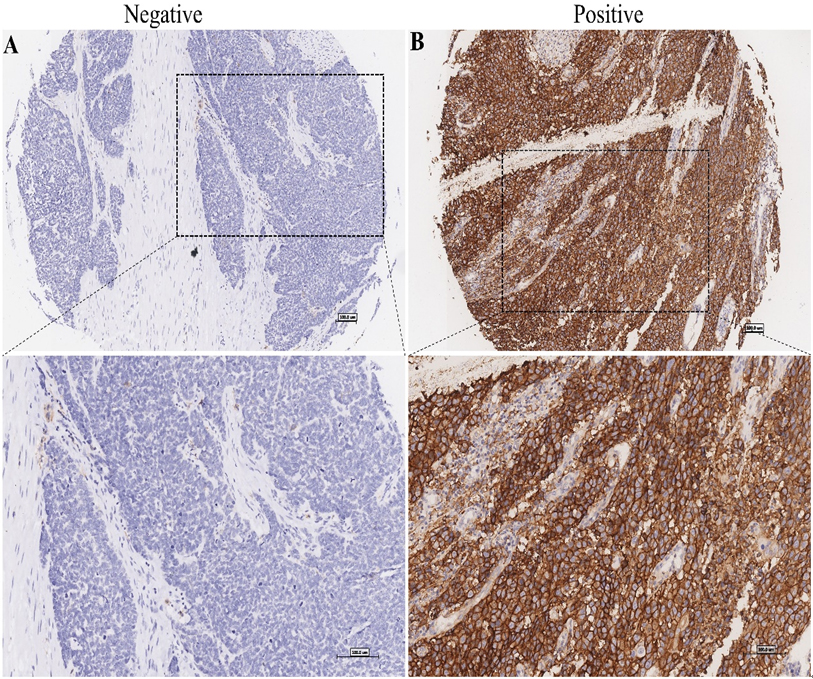

Supplement: Supplementary file 1 — Figure S1 [file JCMM-26-5702-s001.jpg]
